# Supplementary material for: Antimycobacterial Activity of Solid Lipid Microparticles Loaded with Ursolic Acid and Oleanolic Acid: In Vitro, In Vivo, and Toxicity Assessments
Source: Microorganisms. 2024 Oct 25;12(11):2140. doi: 10.3390/microorganisms12112140 (PMC11596724; doi:10.3390/microorganisms12112140)
Supplement: Supplementary file 1 [file microorganisms-12-02140-s001.zip › microorganisms-3194426-supplementary.pdf]

Supplementary Table S1. Effect of treatments on the serum biochemistry parameters of female rats in an acute toxicity.

Group		SGPT (IU/L)	SGOT (IU/L)	ALP (IU/L)	PHO (mg/dl)	BID (mg/dl)	BIT (mg/dl)	ALB (g/dl)	CAA (mg/dl)	PRO (g/dl)	Urea (mg/dl)	Uric Acid (mg/dl)
I	Control	39.57 ± 9.95	111.79 ± 34.69	362.7 ± 88.44	7.96 ± 0.73	0.049 ± 0.01	0.06 ±0.02	4.26± 0.29	8.28 ± 0.33	8.96± 0.2	40.125 ± 8.04	0.59 ± 0.39
II	UA+OA	**56.64 ± 13.47***	110.18 ± 19.45	**500.2 ± 91.96***	**5.8 ± 1.14***	0.04 ± 0.01	0.064 ± 0.02	4.18± 0.37	**10.7 ± 0.35***	8.67± 0.79	35.38 ± 2.56	**1.614 ± 0.44***
V	SLM	39.03 ± 6.09	155.35 ±49.36	322.83 ±52.13	6.99 ± 1.02	0.04 ± 0.01	0.06 ± 0.02	**4.54±0.2***	**11.87 ±0.33***	8.78±0.46	36.1± 3.97	**2.39 ± 1.15***
VI	UA+OA +SLM	39.57 ± 9.95	111.79 ± 34.69	331.0 ± 39.45	7.96 ± 0.73	0.049 ± 0.01	0.06 ±0.02	4.26 ± 0.29	8.28 ± 0.33	8.96 ± 0.52	40.125 ± 8.04	0.59 ± 0.39

The values are represented as mean ± SD (n=6); *p≤0.05, **≤0.01, ***≤0.001, ****≤0.001. SGOT: Serum Glutamic Oxaloacetic Transaminase, SGPT: Serum Glutamic Pyruvic Transaminase, APL: Alkaline Phosphatase, PHO: Phosphate, BID: Direct Bilirubin, BIT: Total Bilirubin, ALB: Albumin, CAA: Calcium, PRO: Protein.

Supplementary Table S2. Effect of dose on lipid profile of rats.

**Group**		**Glucose (mg/dl)**	**Triglyceride (mg/dl)**	**Cholesterol (mg/dl)**	**HDLC (mg/dl)**	**LDL (mg/dl)**
I	Control	79.6 ± 21.61	47.33 ±10.85	79.8 ± 7.01	33.58±4.5	27.62±7.3
II	UA+OA	**95.26±11.76***	36.00 ± 9.43	61 ± 11.87	**49.98±3.55***	**14.77±1.5****
V	SLM	**45.53 ± 28.06***	53.33 ± 15.92	**57.5±11.67***	42.65±2.24	21.72±2.01
VI	[illegible]A+OA+SLM	79.6±21.61	47.33 ±10.85	79.8 ± 7.01	44.48±4.9	22.26±3.0

The values are represented as mean ± SD (n=6); *p≤0.05, **0.01, ****0.0001. HDLC: High Density Lipid Concentration, LDL: Low Density Lipid

Supplimentary Table S3. Evaluation of haematological parameters of rat in acute toxicity study

Group		HB (gm/dl)	RBC (X 10^6 / mm^3)	WBC (X 10^3 /mm^3)	PLT (X 10^3/ mm^3)	PCV (%)	MCV (fl)	MCH (pg)	MCHC (gm/dl)	N (%)	E (%)	L (%)	M (%)
I	Control	14.36±0.43	7.50± 0.23	14.62±2.84	779.6± 113.38	40.2±1.33	53.94±2.42	19.12±0.98	35.52±0.48	10.8±3.03	4.0±3.39	84.6±4.98	0.6±0.55
II	UA+OA	**16.45±0.37***	7.43±0.21	**7.85±2.32***	760.83 ±93.88	**43.97±1.73***	**59.33±3.16***	**22.12±0.87***	**37.42±1.34***	**36.67±4.08***	1.33±0.58	**62.0±3.89***	0.67± 0.52
V	SLM	15.12±1.25	**6.66±0.61***	**7.88±5.96***	7.91±2. 07	40.10±3.33	**60.27±1.03***	**22.70±1.34***	**37.72±2.01***	**66.50±5.75***	1.17±0.75	**31.50±5.24***	0.83±0.75
VI	UA+OA + SLM	16.05±1.95	6.53±1.09	11.62±6.05	12.89± 5.47	40.33±6.33	**62.63±5.72***	**25.47 ± 4.77***	40.38±6.26	**63.83±7.88***	0.67±0.82	**35.17±7.94***	0.33±0.52

Result is represented as mean ± SD (n=6); *p≤0.05, ****≤0.0001. RBC: Red Blood Cells; WBC: White Blood Cells, PLT: Platelets, PCV: Packed cell volume, MCV: Mean Corpuscular volume, MCH: Mean Corpuscular Hemoglobin, MCHC: Mean Corpuscular Hemoglobin Concentration, N: Neutrophils, E: Eosinophils, L: Lymphocytes, M: Monocytes.

Supplementary Figure S1. Effect of UA, OA and HQ on histopathology of liver, kidney, adrenal gland, brain, ovary, lungs, spleen, uterus and heart tissues (Magnification 10X). The areas marked in arrow (⟵) represents mild congestions in the tissue.

Organ	Group I (Control)	Group II (UA)	Group III (OA)	Group IV (HQ)	Group V (SLM)	Group VIII (PCL)
liver	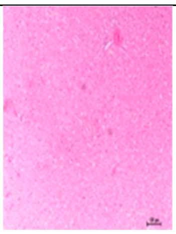	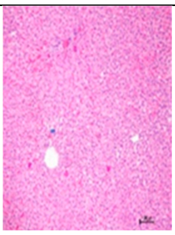	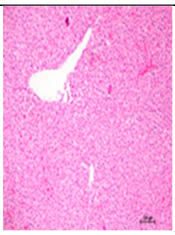		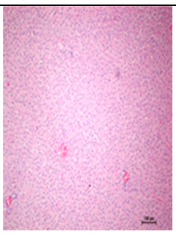	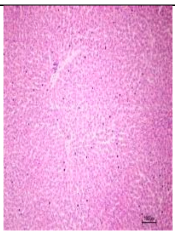
Kidney	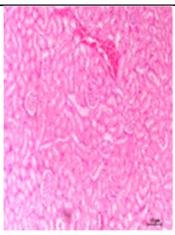	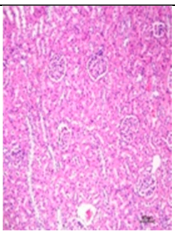	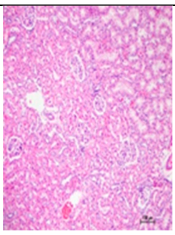	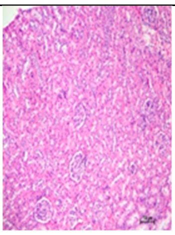	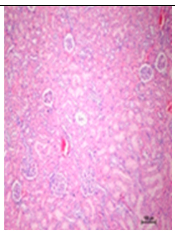	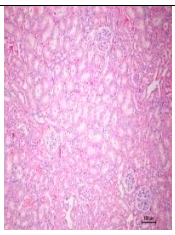
Adrenal gland	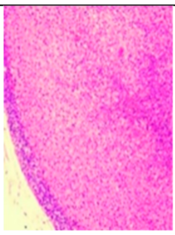	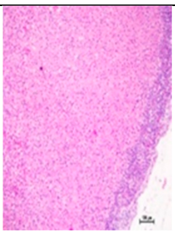	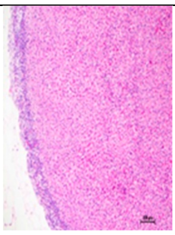	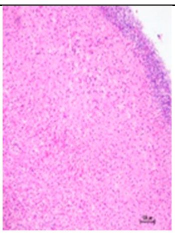	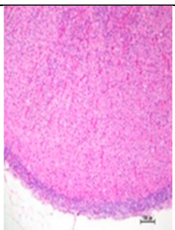	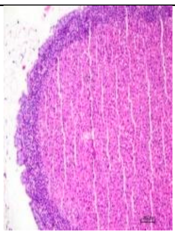
Brain	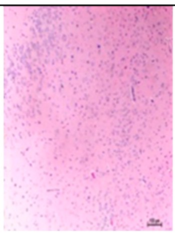	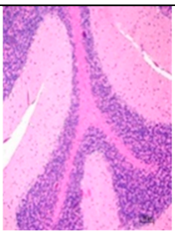	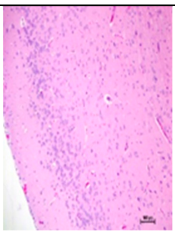	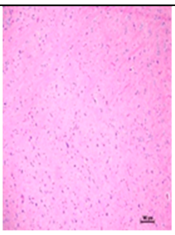	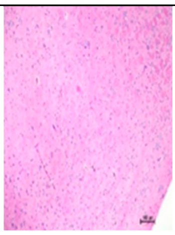	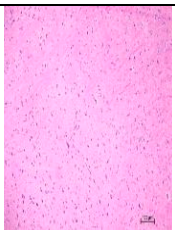
Lungs	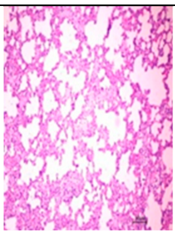	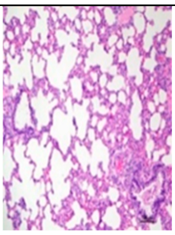	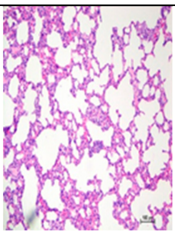	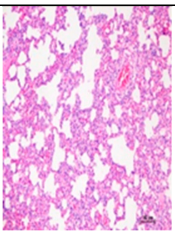	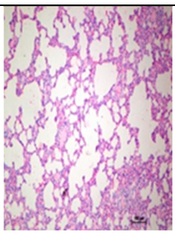	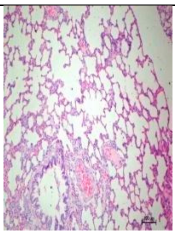

Heart	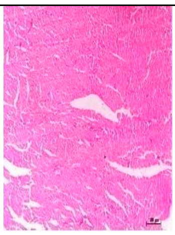	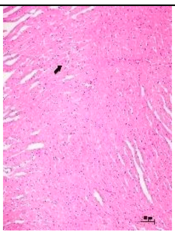	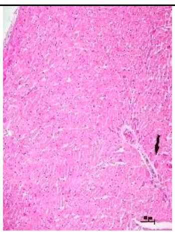	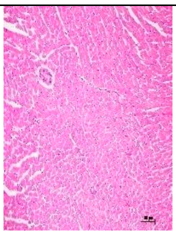	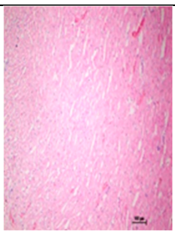	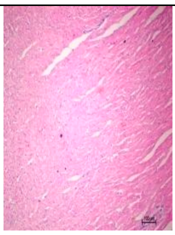
Spleen	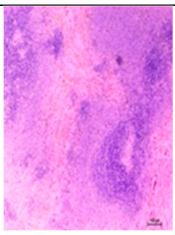	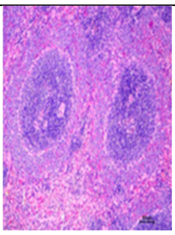	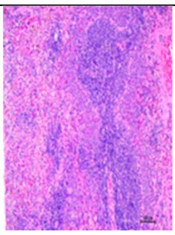	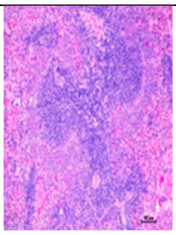	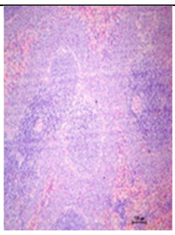	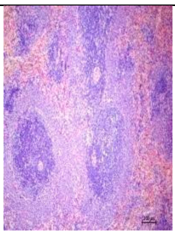
